# Progress in Orthopædics

**Published:** 1898-10-22

**Authors:** 


### PROGRESS IN ORTHOPÆDICS.

(*Continued from page* 33.)

Landerer, after alluding to Calot's method, spoke upon his method of treating inveterate cases of Pott's disease, with abscesses and fistulæ. He had obtained very satisfactory results by attacking the vertebral column from its lateral aspect in the dorsal region by removing ribs, and in the lumbar region by removal of the iliac bone if necessary.

Schede entirely agreed with Hoffa concerning the indications for operation on the Pott's cases. He had constructed an apparatus which allowed him to exercise a very moderate traction of not more than 20 kilos. He applied afterwards a plaster jacket, but putting the head in a separate mould of leather, which could be fixed on to the plaster jacket when the latter was set.

Drehmann had found at the Anatomical Museum at Breslau 37 cases of inveterate spondylitis, of which three alone presented any marked degree of osseous proliferation around the site of disease. From these observations he thought that the probabilities of a spine becoming firmly consolidated by bone after Calot's operation were very slight. He said that they had practised in Mikulicz's clinic 10 redressments, in one of which death followed meningitis. In two of the cases the redressment improved paralysis which existed previously. He thought that redressment was a justifiable operation under certain limited conditions.

König remarked that the pathological ideas which we now possess do not permit us to speak with any certainty as to the probability of the destroyed vertebræ being replaced by osseous tissue, and that being so the results of Calot's method must necessarily be doubtful. For his part he strongly objected to forcible reduction when ankylosis was present.

Kümmell had operated upon a child of eight years, which had presented no sign of suppuration beforehand, and had employed a minimum of mechanical force.

Müller was convinced, after studying osteomyelitic spondylitis for some years, that the cases of the affection which M. Drehmann had quoted were not tuberculous cases but osteomyelitic pure and simple, since tuberculous cases were very rarely accompanied by osteophytes.

In connection with this subject, Vulpius,[2] speaking of the forcible reduction of Pott's disease, recommends that the spinous processes be connected together by small bone periosteal flaps, so that later they may become united by a bony band of union. In applying the plaster of Paris jacket he advises that the patient be suspended after the correction, and the ordinary position be employed in applying these jackets—that is, the vertical. The author reports, however, the death of a five and a half year old boy during this operation, while the jacket was being applied in the manner usual in these cases. After the forcible extension and during the application of the jacket, clonic cramps in the limbs, gnashing of the teeth, and contracted pupils ushered in a collapse that was fatal, and from which the patient could not be resuscitated by removing the jacket and restoring the curvature. A partial post-mortem only was allowed, and disclosed no sufficient cause in any injury of the cord or its meninges.

Ménard,[3] after a careful study of the effect of this method of reduction upon a number of cases in post mortems, and a careful review of the respiratory process as seen in pathological specimens, says that the method must be considered wrong if applied to all forms of Pott's disease, irrespective of their situation and volume. It is impossible to estimate the consequences, and the greatest caution should be maintained in expressing an opinion as to the future usefulness of the method. It is impossible to calculate the accidents which may follow upon this forcible reduction. The result of this procedure upon the tuberculous focus is not known. All that can be said is that in all other parts of the body traumatism aggravates osteo-arthritic tubercular disease, and occasionally gives rise to the formation of abscesses. One cannot be far wrong in predicting grave complications as the result of this treatment. It is the general rule that no new bone is formed in the reparatory process subsequent to tuberculous disease in the vertebra. There is nothing to authorise the view that in this case any bone will be formed to supply the enormous bone defect which is always present. Such bone formation never takes place after tuberculous disease elsewhere. The law is that these defects are filled up, and unions produced by fibrous tissue. As a comment upon this statement of Ménard's, we may point out that in the joints, such as the knee and ankle, synovial membrane is left, which accounts for the union by fibrous tissue. But we have no synovial membrane in the spine, and in most inflammatory processes of the spine the intervertebral discs become replaced by bone and not by fibrous tissue.

[2] Centralblatt fur Chirurgie and Amer. Jour. Med. Sci., July, 1898.
[3] Amer Jour. Med. Sci., May, 1898.

